# Dr. Google to Dr. ChatGPT: assessing the content and quality of artificial intelligence-generated medical information on appendicitis

**DOI:** 10.1007/s00464-024-10739-5

**Published:** 2024-03-05

**Authors:** Yazid K. Ghanem, Armaun D. Rouhi, Ammr Al-Houssan, Zena Saleh, Matthew C. Moccia, Hansa Joshi, Kristoffel R. Dumon, Young Hong, Francis Spitz, Amit R. Joshi, Michael Kwiatt

**Affiliations:** 1 Department of Surgery, Cooper University Hospital, 3 Cooper Plaza, Suite 411, Camden, NJ 08103 USA; 2 Department of Surgery, Perelman School of Medicine, University of Pennsylvania, Philadelphia, PA USA; 3 Department of Surgery, University of Connecticut, Hartford, CT USA; 4 Cooper Medical School of Rowan University, Camden, NJ USA

**Keywords:** Artificial intelligence, Large language models, ChatGPT, Appendicitis, Health literacy, Online medical information

## Abstract

**Introduction:**

Generative artificial intelligence (AI) chatbots have recently been posited as potential sources of online medical information for patients making medical decisions. Existing online patient-oriented medical information has repeatedly been shown to be of variable quality and difficult readability. Therefore, we sought to evaluate the content and quality of AI-generated medical information on acute appendicitis.

**Methods:**

A modified DISCERN assessment tool, comprising 16 distinct criteria each scored on a 5-point Likert scale (score range 16–80), was used to assess AI-generated content. Readability was determined using the Flesch Reading Ease (FRE) and Flesch-Kincaid Grade Level (FKGL) scores. Four popular chatbots, ChatGPT-3.5 and ChatGPT-4, Bard, and Claude-2, were prompted to generate medical information about appendicitis. Three investigators independently scored the generated texts blinded to the identity of the AI platforms.

**Results:**

ChatGPT-3.5, ChatGPT-4, Bard, and Claude-2 had overall mean (SD) quality scores of 60.7 (1.2), 62.0 (1.0), 62.3 (1.2), and 51.3 (2.3), respectively, on a scale of 16–80. Inter-rater reliability was 0.81, 0.75, 0.81, and 0.72, respectively, indicating substantial agreement. Claude-2 demonstrated a significantly lower mean quality score compared to ChatGPT-4 (*p* = 0.001), ChatGPT-3.5 (*p* = 0.005), and Bard (*p* = 0.001). Bard was the only AI platform that listed verifiable sources, while Claude-2 provided fabricated sources. All chatbots except for Claude-2 advised readers to consult a physician if experiencing symptoms. Regarding readability, FKGL and FRE scores of ChatGPT-3.5, ChatGPT-4, Bard, and Claude-2 were 14.6 and 23.8, 11.9 and 33.9, 8.6 and 52.8, 11.0 and 36.6, respectively, indicating difficulty readability at a college reading skill level.

**Conclusion:**

AI-generated medical information on appendicitis scored favorably upon quality assessment, but most either fabricated sources or did not provide any altogether. Additionally, overall readability far exceeded recommended levels for the public. Generative AI platforms demonstrate measured potential for patient education and engagement about appendicitis.

Artificial intelligence (AI) large language models (LLMs) are new online conversational platforms that have revolutionized how information can be sought and obtained. Since their inception in November 2022, these AI platforms have captivated the public by assisting with tasks from the mundane to the more complex. Within one year of launching, ChatGPT alone amassed over 180 million users, and the site receives over 1.5 billion visits per month [1]. As the public continues to evaluate possible uses of AI chatbots, many clinicians have hypothesized and tested different applications of LLMs in healthcare [2, 3].

AI-powered chatbots have recently been tested to assist in clinical decision making, healthcare documentation, and knowledge retrieval [2, 3]. They have also been proposed for use in improving existing patient education materials [4]. Currently available online medical information on a multitude of surgical diseases and procedures has repeatedly been shown to be of variable quality and difficult readability for the public [5–9]. The American Medical Association (AMA) and National Institutes of Health (NIH) recommend that online medical information be written at or below the 6th-grade reading level [10, 11]. The inadequacy of existing online patient-oriented medical information is especially concerning, as low health literacy has been found to be associated with worse patient-centered outcomes and poorer process-oriented surgical outcomes [12]. In this context, AI-powered tools may help bridge this gap, offering readily accessible medical information that can be generated and personalized based on input by the user.

Acute appendicitis is the most common abdominal surgical emergency in the world, with its incidence estimated at 250,000 cases per year in the United States alone and 6.7–8.6% of the population developing appendicitis in their lifetime [13]. If left untreated, acute uncomplicated appendicitis can develop into complicated appendicitis with peritonitis, which can be fatal [13]. The relatively high prevalence of these symptoms and its associated risks in the adult population likely result in many internet searches concerning appendicitis.

The aim of this study was to assess the quality and readability of medical information generated by multiple popular AI chatbots about acute appendicitis, and to evaluate whether AI-generated content can safely be used by the public as a source of online medical information.

## Materials and methods

Four widely used AI chatbots, ChatGPT-3.5 and ChatGPT-4 (OpenAI, San Francisco, California), Bard (Google, Mountain View, California), and Claude-2 (Anthropic, San Francisco, California), were queried to generate content about appendicitis. ChatGPT-3.5, Bard, and Claude-2 are all freely available, while ChatGPT-4 requires a subscription to use. Each chatbot was prompted using four standardized, sequenced questions on September 9th, 2023:

- “Please tell me about appendicitis”
- “Please tell me more”
- “What are the side effects and complications of the treatments?”
- “Please provide a list of sources”

**Fig. 1:**
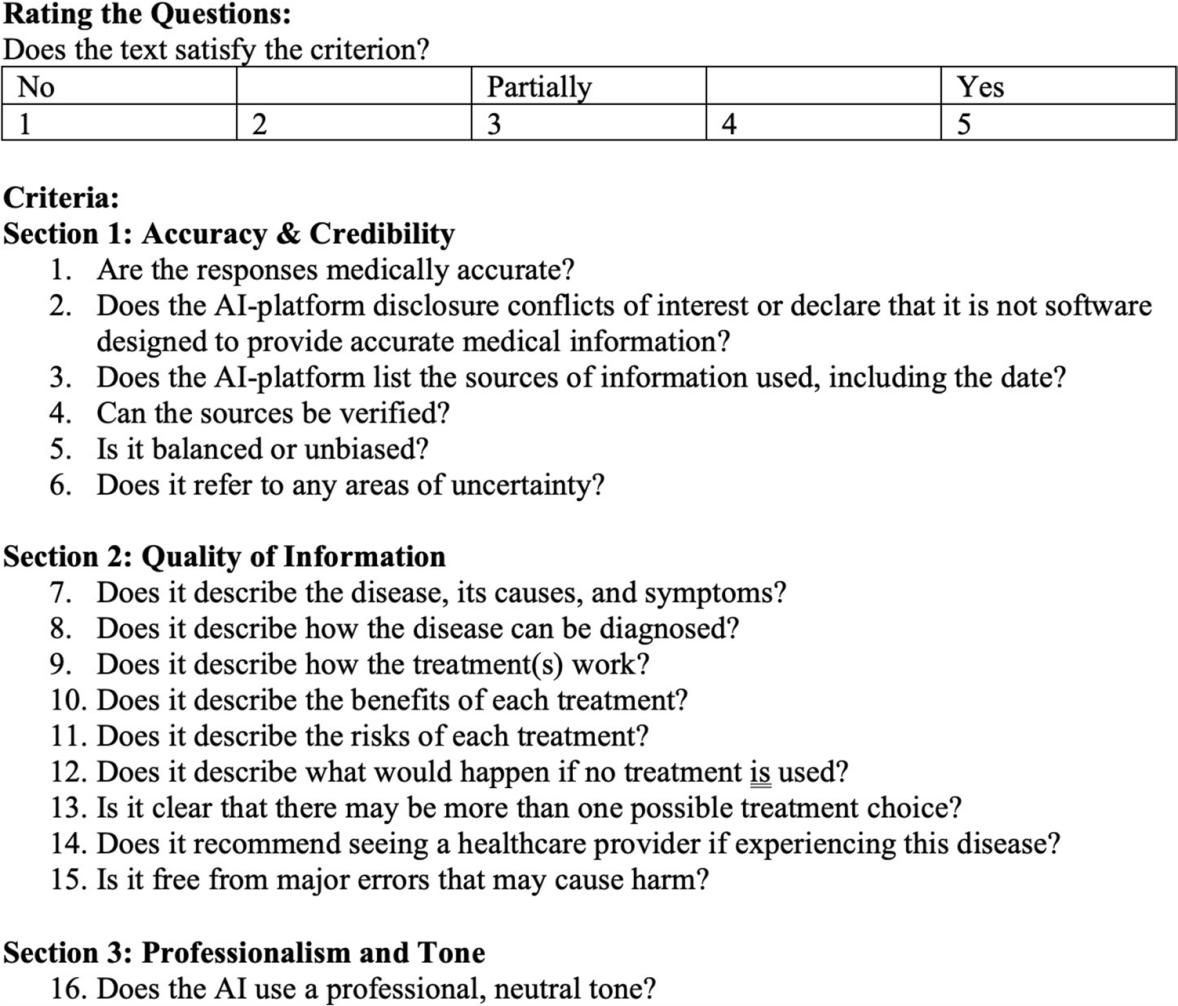
Modified DISCERN instrument

To assess quality and accuracy of the AI-generated text, we utilized a modified DISCERN instrument, which is an existing validated tool used to judge the quality of written consumer health information [14]. Like the original DISCERN instrument, our modified tool includes 16 criteria, each scored on a 5-point Likert scale with a maximum score of 80. Criteria assessed include accuracy, listing disclosures, listing sources, verifying sources, balance and bias, areas of uncertainty, describing the condition, its diagnosis and treatment, benefits and risks of each treatment, risk of nontreatment, recommendation of expert medical opinion, presence of major errors that could cause harm, and overall professionalism/tone (Fig. 1).

Using the modified DISCERN tool, three investigators independently scored the generated texts blinded to the identity of the AI platforms. Prior to scoring, all three investigators comprehensively reviewed UpToDate and the American College of Surgeons information sheet on appendicitis to establish a standard of high-quality medical information for scoring AI-generated content [15, 16]. Inter-rater reliability for quality scores between investigators was calculated using Cohen’s kappa.

These AI-generated texts were then assessed for readability via an online application (https://readable.com, Added Bytes Ltd., Brighton, England) using two validated readability measures: Flesch Reading Ease (FRE) and Flesch Kincaid Grade Level (FKGL) [17, 18]. These readability measures are utilized commonly in health literacy literature [19]. Their formulas are as follows [20]:

$${\text{FRE score}}\, = \,206.835 - 1.015\,\left( {\frac{{{\text{total }}\,{\text{number }}\,{\text{of}}\,{\text{ words}}}}{{{\text{total}}\,{\text{ number }}\,{\text{of}}\,{\text{ sentences}}}}} \right) - \,84.6\left( {\frac{{{\text{total }}\,{\text{number}}\,{\text{ of}}\,{\text{ syllables}}}}{{{\text{total }}\,{\text{number}}\,{\text{ of}}\,{\text{ words}}}}} \right)$$

$${\text{FKGL}}\,\, = \,\,0.39\,\left( {\frac{{{\text{total }}\,{\text{number}}\,{\text{ of}}\,{\text{ words}}}}{{{\text{total }}\,{\text{number }}\,{\text{of }}\,{\text{sentences}}}}} \right)\, + \,11.8\left( {\frac{{{\text{total}}\,{\text{ number}}\,{\text{ of}}\,{\text{ syllables}}}}{{{\text{total}}\,{\text{ number}}\,{\text{ of }}\,{\text{words}}}}} \right)\, - \,15.59$$

The FRE score ranges from 0 to 100 and corresponds to an American educational level, with lower scores indicating more difficult reading material (0–10, extremely difficult/professional level; 10–30, very difficult/college graduate level; 30–50, difficult/college level; 50–60, fairly difficult/10th- to 12th-grade level; 60–70, plain English/8th- to 9th-grade level; 70–80, fairly easy to read/7th-grade level; 80–90, easy to read or conversational English/6th-grade level; 90–100, very easy to read/5th-grade level) [17]. FKGL ranges from 0 to 18, and the score indicates the number of years of education required to comprehend the text [18]. Thus, healthcare education material with FRE scores greater than 80, and FKGL scores of 7 or lower, would correspond with the recommended AMA and NIH readability levels [10, 11].

The primary endpoints of this study were the absolute difference in the quality (modified DISCERN) scores, and the difference in readability scores (FKGL and FRE) between the AI platforms tested. Mean quality scores were recorded and used for analysis. The present study was exempt from institutional review as it did not involve human subjects.

Continuous variables were displayed as means ± standard deviation (SD) or medians with interquartile range if non-parametric. One-way ANOVA with Bonferroni correction was performed to evaluate differences in quality scores between the AI chatbots. A two-sided p-value ≤ 0.05 was considered statistically significant. Statistical analyses were performed using Stata version 18.0 (StataCorp, College Station, TX, USA).

## Results

**Fig. 2:**
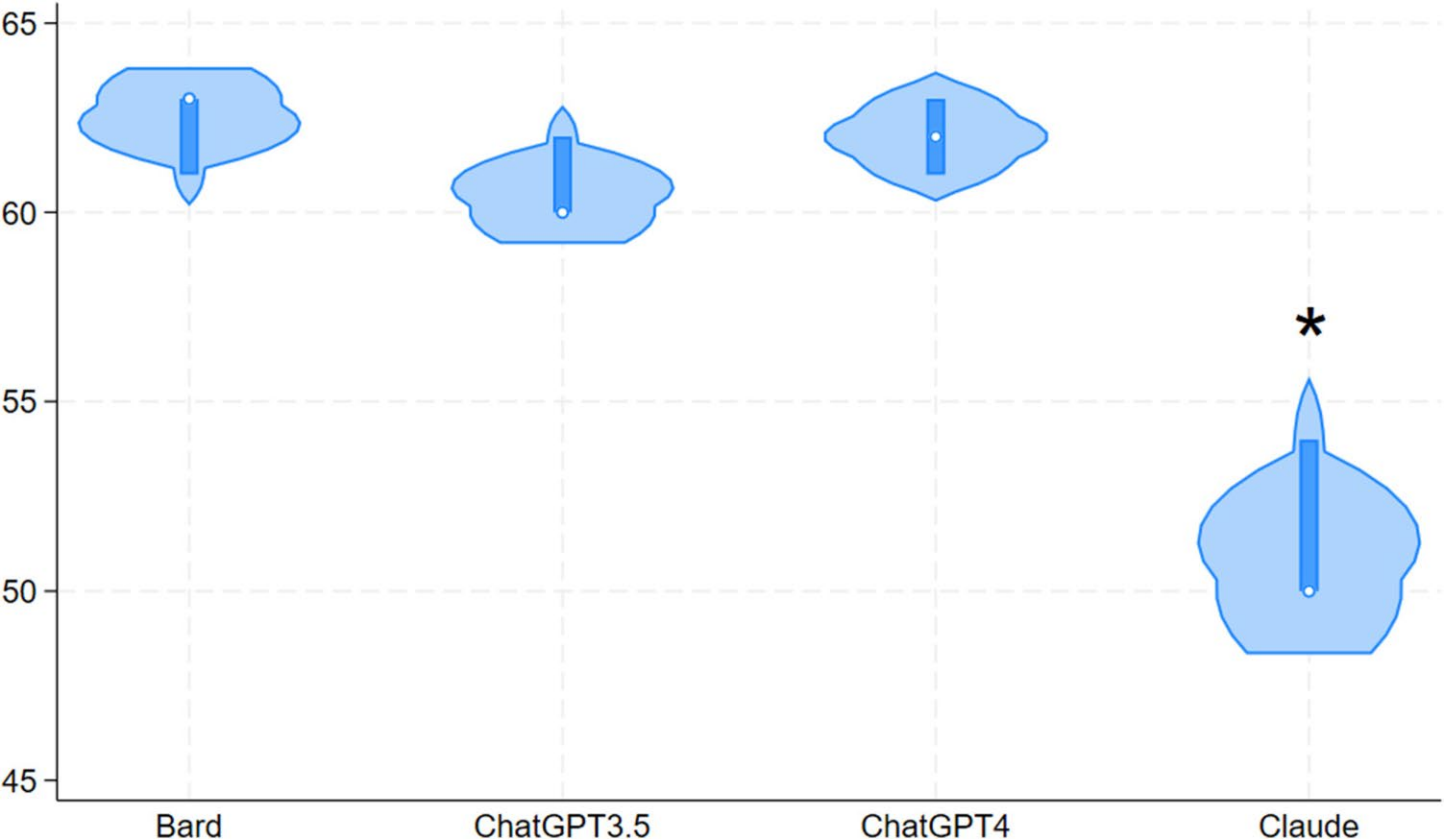
Violin plot of quality scores by artificial intelligence platform. *Indicates significance (*p* < 0.05)

ChatGPT-3.5, ChatGPT-4, Bard, and Claude-2 achieved overall mean (SD) quality scores of 60.7 (1.2), 62.0 (1.0), 62.3 (1.2), and 51.3 (2.3), respectively (Fig. 2). Inter-rater reliability was 0.81, 0.75, 0.81, and 0.72, respectively, indicating substantial to near perfect agreement between investigators. Claude-2 demonstrated a significantly lower mean quality score compared to ChatGPT-4 (*p* < 0.001), ChatGPT-3.5 (*p* < 0.001), and Bard (*p* < 0.001). There was no significant difference in mean quality scores between ChatGPT-3.5, ChatGPT-4, and Bard (Fig. 2).

When comparing specific criteria, Bard was the only AI platform that listed verifiable sources, while Claude-2 provided fabricated sources. ChatGPT-3.5 and GPT-4 did not provide any sources. ChatGPT-4 demonstrated higher quality scores on the bias criterion compared to Bard (*p* < 0.001) and ChatGPT-3.5 (*p* = 0.006), while Claude-2 scored higher than Bard (*p* = 0.006) (Table 1). ChatGPT-4 is the only chatbot that referred to the long-term failure rate of non-surgical treatment of appendicitis. None of the chatbots made any disclosures. However, all four chatbots discussed the risks of not treating appendicitis and provided accurate medical content regarding etiology, symptoms, and treatment with no major factual errors. All chatbots except for Claude-2 advised readers to consult a physician if experiencing symptoms.

**Table 1:** Comparison of mean quality scores (row minus column) regarding bias criterion by artificial intelligence platform

	Bard	Claude-2	ChatGPT-3.5
Claude-2	1.67 (*p* = 0.006)*		
ChatGPT-3.5	1.00 (*p* = 0.102)	− 0.67 (*p* = 0.483)	
ChatGPT-4	2.67 (*p* < 0.001)*	1.00 (*p* = 0.102)	1.67 (*p* = 0.006)*

*indicates significance (*p* < 0.05)

Regarding readability, FKGL and FRE scores of ChatGPT-3.5, ChatGPT-4, Bard, and Claude-2 were 14.6 and 23.8, 11.9 and 33.9, 8.6 and 52.8, 11.0 and 36.6, respectively (Fig. 2). All scores indicated difficulty readability, ranging from a high school student to college graduate reading skill level. Text generated by Bard had the lowest reading difficulty, ranging between 8th and 10th grade reading level.

## Discussion

AI-powered LLMs have been the subject of medical research since their release to the public in November 2022. While healthcare workers have assessed AI for many uses, AI-powered tools, including chatbots, have become widely available to patients. For example, Woebot is a mental health AI-powered chatbot that uses cognitive behavioral therapy techniques to help patients manage anxiety, depression, and other mental health conditions. No such conversational tools exist for surgery yet, but existing heavily developed AI-chatbots such as ChatGPT, Claude-2, and Bard, offer appealing and adaptable choices for patients who use the internet. Recent literature suggests 74% of laypersons use the internet to search about health-related issues [21, 22]. Thus far, multiple studies have assessed the accuracy and quality of medical information generated by AI about bariatric surgery, hepatology, and ophthalmology [23–25]. Other studies assessed the ability of LLMs in clinical settings, for example in diagnosing clinical vignettes, and in medical education [26, 27]. To date, no study has compared the capability of multiple widely available AI platforms to generate medical information about appendicitis, suggesting the present study to be relevant to healthcare systems weighing the value of utilizing these tools for patient education on this common medical condition. In this study, we assessed the quality and readability of medical content about appendicitis produced by four AI platforms: ChatGPT-3.5, ChatGPT-4, Bard, and Claude-2. Claude-2 created content of lower quality than the rest, with significant concerns including fabricated sources and failing to advise readers to seek professional medical care if experiencing symptoms of acute appendicitis. Additionally, all four AI chatbots produced text with difficult readability at the college student reading skill level, which is well beyond the recommended 6th-grade level by the AMA and NIH.

Our findings corroborate prior findings by Samaan et al., who demonstrated good overall quality when assessing ChatGPT-3.5’s information about bariatric surgery [25]. Samaan et al. gathered questions from patient support groups such as Facebook, modified them into suitable prompts, then asked ChatGPT-3.5 a total of 151 questions. The generated text was assessed for accuracy and reproducibility by two bariatric surgeons, with only moderate agreement. In their analysis, ChatGPT-3.5 provided comprehensive responses to over 85% of questions asked about bariatric surgery eligibility, efficacy, procedure options, preoperative preparation, recovery, risks, and lifestyle changes. This indicates generally adequate detail about those procedures for patients seeking additional information before deciding to pursue bariatric surgery as a weight-loss option. We found similar results when prompting the tested LLMs about appendicitis and its etiology, symptoms, and treatment. However, our study interrogated four different AI-platforms, using general questions limited to appendicitis. Unlike bariatric surgery, most appendicitis cases do not require life-long follow-up and support, therefore patients would not usually have additional questions after the acute and recovery phases. Samaan et al. did not assess the readability of generated answers and did not ask the AI-platform to provide a list of sources.

Similarly, Yeo et al. assessed information generated by ChatGPT-3.5 about hepatology topics such as cirrhosis and hepatocellular carcinoma, but from a physician’s point of view [23]. They found that ChatGPT-3.5 offers extensive knowledge, but unlike bariatric surgery, was comprehensive in less than half of the questions asked. Additionally, it lacked the ability to specify decision-making cut-offs (such as MELD-Na scores) and treatment durations and was deficient in its knowledge about regional guideline variations, such as hepatocellular carcinoma screening criteria. They did note that it provided good advice to patients and caregivers. This suggests that AI-platforms in their current form could be more valuable to patients and their caregivers than physicians. Yeo et al.’s grading only assessed accuracy and reproducibility and did not measure readability or ask for sources.

Momenaei et al. assessed ChatGPT-4 generated information about surgical treatment of retinal diseases [24]. Of note, ChatGPT-4 use requires a subscription, unlike GPT-3.5. A list of common questions about definition, prevalence, visual impact, diagnostic methods, surgical and nonsurgical treatment options, postoperative information, surgery-related complications, and visual prognosis were curated. ChatGPT-4 was graded for appropriateness and readability was evaluated using FKGL and FRE scores. Nearly 90% of questions asked had appropriate responses overall. The average FKGL and FRE scores were between 14–15 and 28–35, respectively, indicating difficult or very difficult readability, at the level of a college graduate. Our investigation yielded similar results for ChatGPT-4 when asked about appendicitis, with FKGL and FRE scores of 11.9 and 33.9, respectively. Momenaei et al. did not ask ChatGPT-4 to generate sources, but their results mirror our study’s results, indicating good overall quality in answering questions about surgical specialties with difficult readability, but Yeo et al.’s results show further improvement is needed regarding specific details and cut-offs, especially in non-surgical topics.

Our study highlights the potential these AI-LLMs have in providing patients with accurate information about a common health condition. Three out of the four tested AI-platforms scored favorably, suggesting they are not far away from being useful and a valid option for educating patients. However, progress must be made to make it clear to the reader that those AI-platforms cannot replace trained professionals, with disclosures reminding the reader that they are trained artificial intelligence large language models, with no innate comprehension of the world. This is an overlooked area that has been highlighted recently, as these AI platforms are impressionable by the prompt or information fed through them and can be influenced to produce biased but believable information [28]. Thus, caution must be exercised when using those platforms. None of the aforementioned studies looked into this area when assessing these AI-platforms.

Readability is another area that requires additional progress. The AMA and NIH recommend patient educational material to be at or below a sixth grade reading level to be accessible to the public [10, 11]. As highlighted by our study and Momenaei et al.’s study, these AI platforms produce content that is difficult to read in a wide variety of topics, not just gastrointestinal surgery [24]. This in not unexpected, as these AI-platforms are designed to produce content at the level of an average high school student. Improving this area will facilitate health literacy significantly.

Readability could potentially be improved if the original prompt specified that generated text must be at the 5–6th grade reading level. This has been previously studied. For example, Moons et al. prompted ChatGPT-3.5 and Bard to reduce the reading levels of existing patient educational materials [29]. However, it is unlikely that this is how a lay person may use the AI platforms when searching for information, therefore it is less relevant in our study. Some platforms such as ChatGPT-4 can be programed by the user to adhere to a certain set of preferences. As such, they can be set by the user to only produce text with easy readability. Again, this may not be obvious or known to the average user of the AI platform. In fact, this skill is an emerging area of study coined “Prompt Engineering,” and requires some introduction at a minimum, for the end user to fully utilize the versatile nature of the AI platforms available [30].

Regarding the scoring system used to assess the quality of generated texts, the DISCERN instrument is well validated in the literature, having been used to assess patient-directed healthcare information in hundreds of previously published studies [20]. To adapt DISCERN to evaluate AI-generated text, we modified its criteria to include verifiability of sources and the tone of the produced material. Recent literature has revealed some AI platforms to fabricate otherwise highly believable sources, with convincing titles, authors lists, journals, and even Digital Object Identifier numbers that cannot be verified anywhere in the literature [31]. Moreover, one study has shown the capabilities of AI chatbots in generating authentic-appearing abstracts of fictitious conditions using scientific jargon, and scientists were only able to identify 68% of those abstracts. Thus, it was crucial to include this area in our critical analysis of generated medical content to shed light on this phenomenon [32].

This study was not without limitations. Recommendations and guidelines vary based on region and patient variables such as comorbidities. The AI-platforms tested did not elicit those differences, thus the information generated may not be relevant to all patients. Additionally, these AI-platforms are updated regularly. Minor updates occur without notice. This creates an element of inter-study and temporal variation that cannot be controlled for. One update could generate a technical glitch that would then be remedied in an update. Similarly, one version of the platform could outperform an updated version if the platform becomes adaptable, such that a healthcare worker’s adapted version of the platform becomes more detailed and healthcare oriented, while a layperson’s generated content remains less detailed. Furthermore, researchers may also test those AI-platforms at peak hours and receive different responses compared to off-peak hours, so repeatability may be impacted. Regarding the scoring system employed, DISCERN is well-known and recognized, but there exists an urgent need for a validated AI-oriented scoring system to be developed to ensure comparability between studies. Finally, even after vetting these AI-platforms, it would be imperative that regulators and healthcare administration to closely monitor their output once they are used in real patient interactions.

## Conclusion

The present study compares the medical information generated by four popular AI platforms about acute appendicitis. Our results reveal that ChatGPT-3.5, ChatGPT-4, and Bard produce content of higher quality than Claude-2, although Bard is the only potentially credible chatbot tested, as it was the only platform to list verifiable sources. However, all four platforms produced content too difficult for the public to comprehend, thereby limiting their use in their current form but highlighting gaps for future versions of AI platforms to fill.

## References

[1] Duarte F (2024) Number of ChatGPT users. Exploding Topics. https://explodingtopics.com/blog/chatgpt-users

[2] Shah NH, Entwistle DA, Pfeffer M (2023) Creation and adoption of large language models in medicine. JAMA 330(9):866. 10.1001/jama.2023.14217 37548965 10.1001/jama.2023.14217

[3] Ron L, Kumar A, Chen J (2023) How chatbots and large language model artificial intelligence systems will reshape modern medicine. JAMA Intern Med 183(6):596. 10.1001/jamainternmed.2023.1835 37115531 10.1001/jamainternmed.2023.1835 PMC12049698

[4] Kirchner GJ, Kim RY, Weddle J, Bible JE (2023) Can artificial intelligence improve the readability of patient education materials? Clin Orthop Relat Res 481(11):2260–2267. 10.1097/corr.0000000000002668 37116006 10.1097/corr.0000000000002668 PMC10566892

[5] Rouhi AD, Ghanem YK, Hoeltzel GD et al (2022) Online resources for patients considering hiatal hernia repair: a quality and readability analysis. J Gastrointest Surg 27(3):598–600. 10.1007/s11605-022-05460-4 36127551 10.1007/s11605-022-05460-4

[6] Rouhi AD, Ghanem YK, Hoeltzel GD et al (2022) Quality and readability of online patient information on adolescent bariatric surgery. Obes Surg 33(1):397–399. 10.1007/s11695-022-06385-2 36469204 10.1007/s11695-022-06385-2

[7] Rouhi AD, Ghanem YK, Bader E et al (2023) Online information for incisional hernia repair: what are patients reading? Surgeon 21(4):e195–e200. 10.1016/j.surge.2022.12.002 36588086 10.1016/j.surge.2022.12.002

[8] Rouhi AD, Han JJ, Ghanem YK et al (2022) Quality and readability of online patient information on the left ventricular assist device. Artif Organs 47(6):1029–1037. 10.1111/aor.14479 36478254 10.1111/aor.14479

[9] Rouhi AD, Ghanem YK, Hoeltzel GD et al (2022) Quality and readability assessment of online patient information on cytoreductive surgery and hyperthermic intraperitoneal chemotherapy. J Surg Oncol 127(4):699–705. 10.1002/jso.27143 36394434 10.1002/jso.27143

[10] Weiss BD (2003) Health literacy: a manual for clinicians. American Medical Association Foundation and American Medical Association, Chicago

[11] National Cancer Institute (1994) Clear and simple: developing effective print materials for low literate readers. National Institutes of Health, National Cancer Institute

[12] Trutner Z, Furlough K, Martinez AB et al (2023) Is health literacy associated with surgical outcomes? A systematic review. J Surg Res 291:720–733. 10.1016/j.jss.2023.06.044 37572516 10.1016/j.jss.2023.06.044

[13] Moris D, Paulson EK, Pappas TN (2021) Diagnosis and management of acute appendicitis in adults. JAMA 326(22):2299. 10.1001/jama.2021.20502 34905026 10.1001/jama.2021.20502

[14] Charnock D, Shepperd S, Needham G, Gann R (1999) DISCERN: an instrument for judging the quality of written consumer health information on treatment choices. J Epidemiol Community Health 53(2):105–111. 10.1136/jech.53.2.105 10396471 10.1136/jech.53.2.105 PMC1756830

[15] American College of Surgeons Division of Education (2022) Appendectomy. https://www.facs.org/media/4molizpf/app.pdf. Accessed 10 Sep 2023.

[16] Smink D et al (2023) Management of acute appendicitis in adults. UpToDate. https://www.uptodate.com/contents/management-of-acute-appendicitis-in-adults. Accessed 10 Sep 2023.

[17] Flesch R (1948) A new readability yardstick. J Appl Psychol 32(3):221–233. 10.1037/h0057532 18867058 10.1037/h0057532

[18] Kincaid JP, Fishburne Jr. RP, Rogers RL, Chissom BS (1975) Derivation of new readability formulas (Automated Readability Index, Fog Count and Flesch Reading Ease Formula) for Navy enlisted personnel. Institute for Simulation and Training 56. https://stars.library.ucf.edu/istlibrary/56.

[19] Massie P, Arshad SA, Auyang ED (2024) Readability of American Society of Metabolic Surgery’s patient information publications. J Surg Res 293:727–732. 10.1016/j.jss.2023.09.018 37862852 10.1016/j.jss.2023.09.018

[20] Daraz L, Morrow AS, Ponce OJ et al (2018) Readability of online health information: a meta-narrative systematic review. Am J Med Qual 33(5):487–492. 10.1177/1062860617751639 29345143 10.1177/1062860617751639

[21] Link E, Baumann E (2020) Nutzung von Gesundheitsinformationen im Internet: personenbezogene und motivationale Einflussfaktoren. Bundesgesundheitsblatt—Gesundheitsforschung—Gesundheitsschutz. 63(6):681–689. 10.1007/s00103-020-03144-5 32367207 10.1007/s00103-020-03144-5 PMC8516774

[22] Baumann E, Czerwinski F, Rosset M, Seelig M, Suhr R (2020) Wie informieren sich die Menschen in Deutschland zum Thema Gesundheit? Erkenntnisse aus der ersten Welle von HINTS Germany. Bundesgesundheitsblatt—Gesundheitsforschung—Gesundheitsschutz. 63(9):1151–1160. 10.1007/s00103-020-03192-x 32666180 10.1007/s00103-020-03192-x

[23] Yeo YH, Samaan JS, Ng WH et al (2023) Assessing the performance of ChatGPT in answering questions regarding cirrhosis and hepatocellular carcinoma. Clin Mol Hepatol 29(3):721–732. 10.3350/cmh.2023.0089 36946005 10.3350/cmh.2023.0089 PMC10366809

[24] Momenaei B, Wakabayashi T, Shahlaee A et al (2023) Appropriateness and readability of CHATGPT-4-generated responses for surgical treatment of retinal diseases. Ophthalmol Retina 7(10):862–868. 10.1016/j.oret.2023.05.022 37277096 10.1016/j.oret.2023.05.022

[25] Samaan JS, Yeo YH, Rajeev N et al (2023) Assessing the accuracy of responses by the language model ChatGPT to questions regarding bariatric surgery. Obes Surg 33(6):1790–1796. 10.1007/s11695-023-06603-5 37106269 10.1007/s11695-023-06603-5 PMC10234918

[26] Rao A, Pang M, Kim J et al (2023) Assessing the utility of ChatGPT throughout the entire clinical workflow: development and usability study. J Med Internet Res 25:e48659. 10.2196/48659 37606976 10.2196/48659 PMC10481210

[27] Agarwal M, Sharma P, Goswami A (2023) Analysing the applicability of ChatGPT, bard, and bing to generate reasoning-based multiple-choice questions in medical physiology. Cureus. 10.7759/cureus.40977 37519497 10.7759/cureus.40977 PMC10372539

[28] Giray L (2023) Prompt engineering with ChatGPT: a guide for academic writers. Ann Biomed Eng 51(12):2629–2633. 10.1007/s10439-023-03272-4 37284994 10.1007/s10439-023-03272-4

[29] Moons P, Van Bulck L (2023) Using ChatGPT and Google Bard to improve the readability of written patient information: a proof of concept. Eur J Cardiovasc Nurs. 10.1093/eurjcn/zvad087 37603843 10.1093/eurjcn/zvad087

[30] Meskó B (2023) Prompt engineering as an important emerging skill for medical professionals: tutorial. J Med Internet Res 25:e50638. 10.2196/50638 37792434 10.2196/50638 PMC10585440

[31] Emsley R (2023) ChatGPT: these are not hallucinations—they’re fabrications and falsifications. Schizophrenia. 10.1038/s41537-023-00379-4 37598184 10.1038/s41537-023-00379-4 PMC10439949

[32] Gao C, Howard FM, Markov NS et al (2022) Comparing scientific abstracts generated by ChatGPT to original abstracts using an artificial intelligence output detector, plagiarism detector, and blinded human reviewers. BioRxiv. 10.1101/2022.12.23.52161036561175 10.1101/2022.12.23.521610

